# Engineering *Pseudomonas putida* for the Production of 2,3-Butanediol Isomers and Acetoin

**DOI:** 10.1021/acssynbio.6c00243

**Published:** 2026-04-28

**Authors:** Irene Cano, Isabel de la Torre, Miguel G. Acedos, Jorge Barriuso, José L. Garcia

**Affiliations:** † Department of Biotechnology. Centro de Investigaciones Biológicas Margarita Salas, Consejo Superior de Investigaciones Científicas (CIB-CSIC), 28040 Madrid, Spain; ‡ Advanced Biofuels and Bioproducts Unit, Department of Energy. Centro de Investigaciones Energéticas, 54446 Medioambientales y Tecnológicas (CIEMAT). 28040 Madrid, Spain

**Keywords:** *Pseudomonas putida* KT2440, 2,3-butanediol, acetate, furfural, metabolic engineering, bioreactor optimization

## Abstract

The strain *Pseudomonas putida* KT2440 has been genetically modified to synthesize 2,3-butanediol (2,3-BDO). To achieve this goal, we have used as a host the *P. putida* KT2440 *acoB::mini-Tn5* mutant unable to mineralize acetoin and 2,3-BDO. This strain has been further engineered by overexpressing in the pIZ2 plasmid, using the inducible promoter *P*_*trc*_, the *budABC* operon from *Klebsiella oxytoca*, which encodes the three enzymes required to transform pyruvate into 2,3-BDO. Flask and bioreactor experiments were carried out for the optimization of the 2,3-BDO production using acetate or glucose as substrates, which was highly dependent on the initial substrate concentration and the optimal oxygen supply. Using glucose in a fed-batch bioreactor enabled the highest diol (i.e., 2,3-BDO stereoisomers and acetoin) yield, attaining a concentration of 16 g/L, while the maximum production of diols in a fed-batch using acetate as a substrate was 1 g/L. The recombinant *P. putida* KT2440 *acoB::mini-Tn5* (pIZ2Bud) showed a high tolerance to furfural, a contaminant produced after lignocellulose pretreatment, which can facilitate the production of 2,3-BDO from this residue. This is the first report on *P. putida* for the production of 2,3-BDO, which may contribute to facilitating the use of this chassis at an industrial scale.

## Graphical abstract

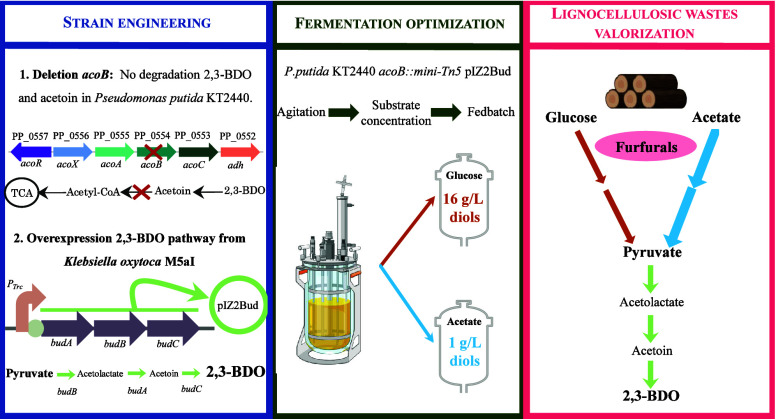

## 1 Introduction

The C4 molecules 2,3-butanediol (2,3-BDO) and 3-hydroxybutanone (acetoin) serve as versatile platform chemicals with a wide range of industrial uses.^1^ 2,3-BDO is a nontoxic liquid valued for applications such as fuel production, bulk chemical manufacturing, food formulation, antifreeze formulations, and as an intermediate in bioplastic synthesis.^2^ The chemical synthesis of 2,3-BDO renders a racemic mixture of the three existing optical isomers (*meso*-2,3-BDO, (*2R,3R*)-2,3-BDO, and (*2S,3S*)-2,3-BDO).^3^ On the other hand, 2,3-BDO is biologically produced as an overflow metabolite of the Embden–Meyerhof–Parnas (EMP) pathway by several Gram-positive and Gram-negative bacteria. Depending on the microorganism, a specific 2,3-BDO isomer can be accumulated in the fermentation broth.^4^ *Klebsiella pneumoniae* is one of the strains producing *meso*-2,3-BDO with high purity and productivity,^5^ whereas the production of the *R*-2,3-BDO isomer has been studied in *Paenibacillus polymoxa*.^6^ In addition, several non-natural producer bacteria and yeasts have been engineered for 2,3-BDO production, including *Escherichia coli*, *Lactobacillus lactis*, *Corynebacterium glutamicum*, and *Saccharomyces cerevisiae*.^7–12^ The biosynthetic pathway for 2,3-BDO production involves the conversion of pyruvate through the catalytic activities of three central enzymes: (i) α-acetolactate synthase (ALS, EC 4.1.3.18), (ii) α-acetolactate decarboxylase (ALDC, EC 4.1.1.5), and (iii) butanediol dehydrogenase (BDH, EC 1.1.1.76), although in some organisms this reaction can also be carried out by acetoin reductase (ACR, EC 1.1.1.4).^10^ The pathway is initiated by ALS, which catalyzes the formation of α-acetolactate from two molecules of pyruvate. Subsequently, α-acetolactate is transformed into acetoin by the ALDC enzyme. The final step involves the reduction of acetoin to 2,3-BDO by BDH. 2,3-BDO can be reversibly converted into acetoin, coupled to the oxidation of NADH. The stereospecificity of the BDH determines the product: some variants yield *meso*-2,3-BDO^13^ and others yield *R*-2,3-BDO.^6^

2,3-BDO synthesis typically requires oxygen-limited environments, because in fully aerobic settings the pyruvate dehydrogenase complex predominantly converts pyruvate into acetyl-CoA rather than channeling it toward 2,3-BDO formation. In addition, under aerobic conditions, α-acetolactate can spontaneously decarboxylate, leading to the formation of diacetyl.^14^

The 2,3-BDO pathway primarily contributes to avoiding strong drops in intracellular pH and helps maintain the cellular NADH/NAD^+^ redox balance.^15^ In specific microorganisms, the induction of ALS expression serves to prevent the excessive accumulation of acetic acid by channeling pyruvate flux toward the formation of 2,3-BDO, effectively regulating the intracellular pH environment.^16,17^

On the other hand, when glucose is depleted in the growth medium, due to the reversibility of BDH, NADH can be regenerated. Therefore, at the end of the fermentation process, the precise management of glucose concentration and oxygen availability is critical to control the 2,3-BDO/acetoin ratio.^4^

There are several microorganisms that can produce and metabolize 2,3-BDO at the same time, using it as an energy and carbon source when glucose is depleted, to maintain cellular metabolism. This is the case for *Bacillus subtilis* and *Bacillus licheniformis*, which have the *aco* operon, which is responsible for 2,3-BDO and acetoin catabolism.^18^ However, the *aco* operon is also present in other bacteria that do not produce 2,3-BDO, such as *Pseudomonas putida* KT2440, to allow them to use environmental 2,3-BDO and acetoin as carbon and energy sources.^19–21^ *P. putida* KT2440 is widely used in biotechnology because it can process many substrates and withstand organic solvent stress (Wackett, 2003; Belda et al., 2016). KT2440 has been proposed as a chassis for industrial biotransformations because of its nonpathogenic nature, its versatile metabolism, robust redox metabolism, rapid growth on different substrates, its ability to tolerate a wide range of stresses, and the availability of mutants and genetic modification tools.^22^

However, despite *P. putida* KT2440 being considered an excellent cell factory to produce many biobased compounds, it has not been used until now as a platform to produce 2,3-DBO because of the presence of the degrading *aco* cluster.^23,24^ Accordingly, this work focused on developing a *P. putida* KT2440 variant capable of producing 2,3-BDO with high efficiency. This chassis can be of special interest for the production of 2,3-BDO using lignocellulosic hydrolysates, where high concentrations of sugars and acetate are present, and where toxic compounds such as furfural are generated during the pretreatment processes.^25^ To achieve this goal, an *acoB* mutant of *P. putida* was transformed with an expression vector containing the *budABC* operon from *Klebsiella oxytoca*, which encodes the enzymes ALDC (*budA*), ALS (*budB*), and BDH (*budC*). The recombinant *acoB* mutant of *P. putida* harboring the operon *budABC* was capable of converting glucose and acetate into 2,3-BDO. Optimization of 2,3-BDO formation was carried out using both batch and fed-batch bioreactor setups.

## 2 Materials and Methods

### 2.1 Bacterial Strains, Plasmids, and Media Culture

Bacterial strains, their respective genotypes, plasmids, and oligonucleotides used in this study are listed in Table 1. *E. coli* DH10B and *K. oxytoca* M5aI were grown aerobically in Luria–Bertani (LB) complex medium (Gibco Bacto Yeast extract, Fisher Scientific),^26^ at 37 °C in cultures of 10 mL in 50 mL Erlenmeyer flasks at 200 rpm (New Brunswick Innova 44/44R, Germany). *P. putida* KT2440 was also cultured aerobically in LB at 30 °C as 10 mL cultures in 50 mL Erlenmeyer flasks on a rotary shaker at 200 rpm. For long-term storage, *E. coli*, *K. oxytoca*, and *P. putida* strains were conserved at −80 °C in 30% (w/v) glycerol and were streaked out on LB solid medium with antibiotics if necessary for cultivation (kanamycin (Km) 50 mg/L and gentamicin (Gm) 10 mg/L).

**Table 1:** Strains, Plasmids, and Primers^a^

**strains**	**genotype and/or description**	**reference**
*Klebsiella oxytoca*		
M5aI	Isolated from soil and free of capsular polysaccharides.	DSM 3539
*Escherichia coli*		
DH10B	F′, *mcrA, Δ(mrr, hsdRMS-mcrBC), Φ80lacZΔM15, ΔlacX74, deoR, recA1, araD139, Δ(ara-leu)7697, galU, galK, rpsL* (Sm^R^ *), endA1, nupG*	Invitrogen
DH10B (pIZ2Bud)	DH10B carrying pIZ2Bud plasmid, Gm^R^	this work
*Pseudomonas putida*		
KT2440	Rf^R^, TOL plasmid-cured, spontaneous restriction-deficient derivative of *P. putida* mt-2	^45^
KT2440-*acoB::mini-Tn5*	*acoB* mutant of KT2440 (PP_0554) obtained by insertion of mini-Tn5 transposon	*Pseudomonas* reference culture collection (Estación Experimental del Zaidín, CSIC)^46^
KT2440-*acoB::mini-Tn5* (pIZ2Bud)	*P. putida* KT2440-*acoB::mini-Tn5* carrying pIZ2Bud plasmid, Gm^R^	this work

**plasmid**	**description**	**reference**
pTOPO (pCRBlunt II-TOPO)	Km^R^, *P*_*lac*_ promoter operator, *lacZα*-*ccdB*. pUC origin	Thermo Fisher
pTOPOBud	Km^R^. pTOPO harboring *budABC* operon from *K. oxytoca* in a fragment of 3200 bp	this work
pIZ2	Gm^R^. pIZ1016 derivative carrying a modified polylinker. pBBR1MCS-5 broad-host range cloning vector.	^47^
pIZ2Bud	Gm^R^. pIZ2 harboring *budABC* operon from *K. oxytoca* in a fragment of 3200 bp	this work

**primers**	**description**	**sequence**
BudA Fw *Eco*RI	amplification of the fragment upstream bud cluster	cccccGAATTCtgacctaaggaggtaaataatgaatgaaccattctgttgaatgctc
BudC Rv *Hin*dIII	amplification of the fragment downstream bud cluster	cccccAAGCTTttagttaaataccatgccgccatcg
Bud Sec1	sequencing primer	gagattctcgtggataatcaacatc
Bud Sec2	sequencing primer	gccatgtggaataacggtaacgc

a Restriction enzymes are shown in capital letters.

To produce 2,3-BDO, we used M9 2× minimal medium (pH 7) supplemented with trace elements solution, 3 g/L of yeast extract, and different amounts of acetate or glucose as carbon sources.^27^ This medium was named M9YE.

It is worth mentioning that the culture medium used to produce 2,3-BDO is not a true minimal medium since it includes yeast extract to reduce the lag phase and facilitate the growth of the strains. Yeast extract is only used to produce biomass, since in the absence of glucose, the strains cannot produce 2,3-BDO (data not shown). Therefore, although the total carbon used in the assays would consider both glucose and yeast extract, in fact, the calculations of the 2,3-BDO yields in this work are performed only based on glucose consumption.

To determine if *P. putida* can use 2,3-BDO or acetoin as sole carbon sources, we cultured the wt strain and the *acoB* mutant in M9 minimal medium at pH 7 ^27^ with 0.2% of 2,3-BDO or 0.2% of acetoin.

The tolerance to furfural (Merck, Germany) was determined in 50 mL flasks by culturing *P. putida* and *E. coli* cells in 10 mL of M9YE 2× minimal medium at pH 7 and supplemented with trace elements solution, 3 g/L of yeast extract, and 0.2% glucose as a carbon source by adding increasing furfural concentrations up to 3 g/L. OD at 600 nm was used to estimate cell growth after 24 h of culture (30 °C and 200 rpm) (New Brunswick Innova 44/44R, Germany).

### 2.2 Recombinant DNA Procedures

*P. putida* KT2440 cells grown overnight in 10 mL of LB were made electrocompetent by centrifugation (3000*g*, 20 min, 4 °C) and washing (5×) with 300 mM sucrose before resuspension. Aliquots (100 μL of sucrose solution) were mixed with 100 ng of plasmid and electroporated at 25 μF, 2.5 kV, and 200 Ω in 2 mm cuvettes (Bio-Rad, US). Then, cells were resuspended in 900 μL of LB and incubated for 1 h at 30 °C. Dilutions were plated on LB agar (1.5%) with antibiotics.^28^

### 2.3 Plasmid Construction

Heterologous expression of the metabolic pathway of 2,3-BDO was achieved by expressing the operon *budABC* from *K. oxytoca* M5aI in a plasmid that replicates in both *E. coli* and *P. putida*. To build this plasmid, genomic DNA from *K. oxytoca* M5aI was extracted,^29^ washed with 70% ethanol by mixing and centrifuging (2 min, 14,000*g*), and then dried in a SpeedVac.

The operon *budABC* was amplified from the genomic DNA of *K. oxytoca* M5aI using the primers BudA Fw *Eco*RI and BudC Rv *Hin*dIII (Table 1). The resulting PCR fragment was integrated into the vector pCRBlunt II-TOPO (Thermo Fisher, US) to render the pTOPOBud plasmid that was sequenced using the primers in Table 1. Then, the *bud* operon was transferred to the plasmid pIZ2,^27^ and the pIZ2Bud plasmid was sequenced using the primers in Table 1. Finally, this plasmid was electroporated into *P. putida* KT2440,^28^ generating the strain *P. putida* KT2440 (pIZ2Bud).

### 2.4 Acetoin and 2,3-BDO Production

Starter cultures of the strains were grown overnight in LB medium supplemented with gentamicin (10 mg/L). Cells were harvested by centrifugation (4500*g*, 15 min, 4 °C), rinsed with sterile saline, and then inoculated into the production medium to an initial OD_6_00 of 0.1. For 2,3-BDO and acetoin synthesis by *P. putida* KT2440 (pIZBud), 50 mL of M9YE 2× medium enriched with 3 g/L yeast extract, 20 g/L glucose, and gentamicin (10 mg/L) were used in 250 mL Erlenmeyer flasks. Cultivations were conducted at 30 °C with shaking at 200 rpm. Induction of the *bud* operon was achieved by adding IPTG at various concentrations (0.01, 0.02, 0.05, 0.1, or 0.5 mM) 2 h after inoculation.

Batch fermentations for 2,3-BDO and acetoin production were conducted in a 0.5-L bioreactor (Infors Multifors 2, Switzerland) operated with 0.3 L of M9YE 2× medium supplemented with 3 g/L yeast extract and 10 mg/L gentamicin. To evaluate the effect of the mixing intensity, cultures were supplied with 20 g/L glucose and stirred at 200, 400, 600, or 800 rpm. The influence of substrate availability was examined by varying the initial glucose concentration (20–120 g/L) while maintaining agitation at 400 rpm. All bioreactor runs were performed at 30 °C, with the pH regulated at 6.8 through the addition of 2 M NaOH or 2 M HCl. Aerobic conditions were maintained by supplying air at 1 vvm. Induction of the *budABC* operon was achieved by adding 0.1 mM IPTG 2 h after inoculation.

Fed-batch experiments were carried out in duplicate in the same 0.5-L system containing 0.3 L of M9YE 2× medium with 3 g/L yeast extract, 40 g/L glucose, and 10 mg/L gentamicin at the start. Cultures were maintained at 30 °C, with pH controlled at 6.8 via automated addition of 2 M NaOH or 2 M HCl, and aeration set to 1 vol% with agitation at 400 rpm. The *budABC* operon was induced with 0.1 mM IPTG after 2 h. When the initial glucose was exhausted, a 30 mL feeding solution was added to restore glucose and yeast extract levels to 40 and 3 g/L, respectively. The feed, delivered in pulses, contained 400 g/L of glucose and 30 g/L of yeast extract. This strategy supported sustained biomass formation, continuous glucose uptake, and ongoing diol production.

### 2.5 Analytical Methods

Substrate and product concentrations throughout the fermentation were quantified by HPLC. Briefly, 1 mL samples were collected and centrifuged (14,000*g*, 1 min, 4 °C), and the supernatants were passed through 0.2-μm PETC filters (Thermo Fisher, US) prior to analysis. Metabolite separation was performed on an Agilent 1200 HPLC system equipped with an Aminex HPX-87C column (1300 mm × 7.8 mm; 9 μm, Bio-Rad). The eluent consisted of 5 mM H_2_SO_4_, with the column maintained at 70 °C and operated at a flow rate of 0.4 mL/min for a total run time of 40 min. A 15-μL injection volume was used, and detection was carried out with a refractive index detector (RID; G7162A, Agilent 1260). Quantification of each compound relied on external calibration curves constructed at four concentration points. In this study, the term ‘diols’ refers to the combined concentrations of acetoin and 2,3-BDO. Although acetoin is not chemically a diol, its reversible conversion to 2,3-BDO leads many authors to report the sum of both metabolites when assessing 2,3-BDO production.^30–33^

OD 600 nm was determined in a spectrophotometer (Shimadzu UV mini 1240), and the g/L was calculated with the following equation:

$$\mathrm{biomass}\left(g/L\right)=0.5\times {\mathrm{OD}}_{600\mathrm{nm}}$$ 1

Yield, selectivity, and productivity were calculated as follows:

$$\mathrm{yield}(\%)=\left(\frac{\mathrm{diols}\left(g/L\right)}{\mathrm{carbon}\;\mathrm{source}\left(g/L\right)}\right)\times 100$$ 2

$$\mathrm{selectivity}=\frac{\mathrm{diols}\left(g/L\right)}{\mathrm{carbon}\;\mathrm{source}\;\mathrm{consumed}\left(g/L\right)}$$ 3

$$\mathrm{productivity}=\frac{\mathrm{diols}\left(g/L\right)}{\mathrm{time}(h)}$$ 4

The maximum theoretical yield was calculated based on the stoichiometry of the relevant metabolic pathways. Although *P. putida* metabolizes glucose through a unique cyclic pathway involving the Entner-Doudoroff, Embden-Meyerhof-Parnas, and pentose phosphate pathways, we can assume the following stoichiometric reaction to calculate the maximum yield for glucose conversion to 2,3-BDO:

$$\mathrm{Glucose}+2\mathrm{ADP}+2\mathrm{Pi}+{\mathrm{NAD}}^{+}\to 2,3-\mathrm{BDO}+2{\mathrm{CO}}_{2}+2\mathrm{ATP}+\mathrm{NADH}+H^{+}$$

Therefore, the maximum 2,3-BDO yield using glucose as the substrate will be 0.5 g/g of glucose.

The metabolic transformation of acetate to 2,3-BDO requires initial activation to acetyl-CoA through the ATP-dependent ACK/PTA pathway. To yield the necessary precursors, four acetyl-CoA molecules are processed via the glyoxylate bypass into two pyruvate molecules, generating two NADH molecules and two FADH_2_. Of these, one NADH supports 2,3-BDO production, while the oxidative phosphorylation of the remaining NADH and FADH_2_ provides the 4 ATP molecules essential for the pathway’s energy balance. This leads to the following stoichiometry. The resulting overall stoichiometry is as follows:

$$4\mathrm{acetate}+2H_{2}O+4\mathrm{ATP}+{\mathrm{NAD}}^{+}+2\mathrm{FAD}\to 2,3-\mathrm{BDO}+4{\mathrm{CO}}_{2}+\mathrm{NADH}+2{\mathrm{FADH}}_{2}+4\mathrm{ADP}+4\mathrm{Pi}+H^{+}$$

Thus, the maximum 2,3-BDO yield using acetate as the carbon source will be 0.38 g/g of acetate.

## 3 Results

### 3.1 Engineering *P. putida* for Acetoin and 2,3-BDO Production

Since *P. putida* KT2440 is able to grow on acetoin or 2,3-BDO as carbon and energy sources using the *acoRXABC* operon, it cannot be used as such to produce 2,3-BDO (Figure S1). Therefore, we tested the growth of *P. putida* KT2440-*acoB::mini-Tn5* mutant and the wild type (wt) (Table 1) on 0.2% of acetoin or 0.2% of 2,3-BDO. As expected, this *acoB* mutant was not able to grow on acetoin or 2,3-BDO, while the wt did (Figure S2). Thus, this mutant was used to carry out the production of 2,3-BDO. It is worth mentioning that the transposon insertion in the *acoB* gene will affect the transcription of at least the *acoC* gene, and perhaps the transcription of the *adh* gene, although it is not clear if the *adh* gene can also be transcribed independently by its own promoter. Moreover, we cannot discard the possibility that the insertion might also affect the transcription of the *acoA* and *acoX* genes. In addition, we have to consider that the presence of acetoin in the medium will induce the expression of the *acoA* and *acoX* genes as suggested.^21^ We must also consider that in the presence of glucose, the expression of the *aco* genes can be repressed, as demonstrated.^21^ In any case, we have confirmed that the absence of a functional *acoB* gene is enough to avoid the metabolization of acetoin^21^ and that the insertion does not alter the growth of the *acoB* mutant in glucose when compared to the wt strain (Figure S2).

To channel the carbon source from pyruvate to 2,3-BDO, we constructed a plasmid harboring the operon *budABC* from *K. oxytoca* (Table 1). The resulting plasmid pIZ2Bud, which expresses the *budABC* operon under the control of the IPTG-inducible *P*_*trc*_ promoter, was initially cloned into *E. coli* DH10B. The resulting *E. coli* DH10B (pIZ2Bud) strain was able to produce diols (acetoin plus 2,3-BDO) in flasks using glucose in the presence of IPTG, confirming the full functionality of the *budABC* operon, producing 18 g/L of diols after 48 h of fermentation using 60 g/L of glucose (Figure S3). Taking advantage of the fact that pIZ2Bud also replicates in *P. putida*, we created the recombinant *P. putida* strain KT2440-*acoB::mini-Tn5* harboring the pIZ2Bud plasmid. This strain was able to produce 2 g/L of diols in shake flask experiments using 20 g/L of glucose and 0.5 mM of IPTG as an inducer, after 48 h at 30 °C and 200 rpm. The production of 2,3-BDO was studied by HPLC, confirming the production of *meso*-2,3-BDO (25.9 min) and *R*-2,3-BDO (27.1 min) (Figure S4). It is interesting to note that in this chromatogram, no significant amounts of acetate, succinate, lactate, ethanol, or other byproducts are observed.

On the other hand, different concentrations of IPTG were tested to find the optimal value to induce the *budABC* operon of the pIZ2Bud plasmid. Figure 1 shows the influence of IPTG concentration on diol production by *P. putida* KT2440-*acoB::mini-Tn5* (pIZ2Bud), suggesting that the minimum IPTG concentration required to obtain the maximum yield of 2,3-BDO is 0.05 mM. Higher concentrations of IPTG do not improve diol production. A concentration of 0.1 mM IPTG was selected as the best induction condition for the next experiments and the possibility to operate under fed+batch conditions, where the IPTG concentration can be diluted.

**Figure 1:**
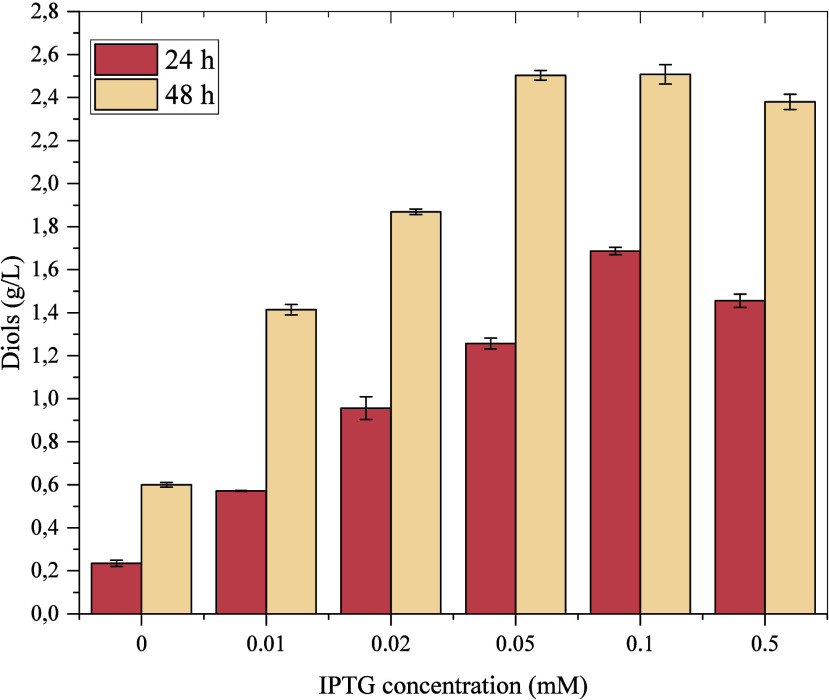
Diol production in flasks using *P. putida* KT2440 *acoB::mini-Tn5* (pIZ2Bud) with different IPTG concentrations in M9YE 2× medium using glucose as a carbon source at 0.2% concentration. The cells were grown with orbital agitation. The samples were collected at 24 h (pink bars) and 48 h (yellow bars), and the production of diols (both acetoin and 2,3-BDO) was calculated by HPLC analysis.

### 3.2 Tolerance of *P. putida* Recombinant to Acetate and Furfural

Saccharified lignocellulosic waste can be an alternative source of glucose for producing 2,3-BDO. However, the pretreatment of this type of waste can yield, along with sugars, significant amounts of potentially toxic compounds such as furfural and acetate, although the latter could also be a carbon source for growth and diol production. In this sense, we determined the tolerance to furfural of the *P. putida* KT2440-*acoB::mini-Tn5* (pIZ2Bud) strain compared to the *E. coli* DH10B (pIZ2Bud) strain. Figure 2 shows that *P. putida* is able to grow in the presence of higher concentrations of furfural than *E. coli*, tolerating up to 2.5 g/L of furfural.

**Figure 2:**
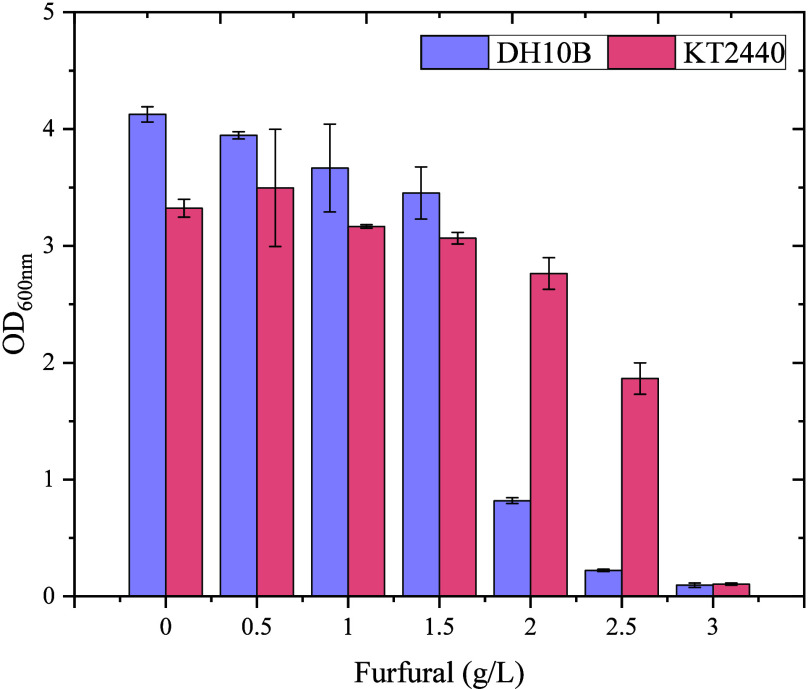
Tolerance to increasing furfural concentration of *E. coli* DH10B (pIZ2Bud) and *P. putida* KT2440 *acoB::mini-Tn5* (pIZ2Bud) in 2,3-BDO production medium M9YE 2× supplemented with glucose and yeast extract. Furfural was added from the beginning of the culture at indicated concentrations, and the cells were grown with orbital agitation. The growth of each strain was measured after 24 h by OD at 600 nm, with the growth of DH10B represented by purple bars and the KT2440 strain by pink bars.

On the other hand, we have determined that acetate is somewhat toxic to *P. putida* KT2440-*acoB::mini-Tn5* (pIZ2Bud) at concentrations above 5 g/L, since although it can grow even using acetate at 10 g/L, it shows a long lag phase for adaptation (data not shown).

### 3.3 Optimization of 2,3-BDO Yield in Bioreactors

To optimize the yield of 2,3-BDO production in *P. putida* KT2440-*acoB::mini-Tn5* (pIZ2Bud), we tested different controlled culture conditions in bioreactors. Initially, four different agitation rates were tested using 20 g/L of glucose as a carbon source in batch cultures. Figure 3 shows that the best biomass production was obtained at 800 rpm, and the maximum concentration of diols (5 g/L) was achieved at 400 rpm. We have observed that glucose is not fully consumed at 200 rpm, and the rate of glucose uptake increases with agitation. This trend aligns with biomass evolution but not with 2,3-BDO production, as mentioned above.

**Figure 3:**
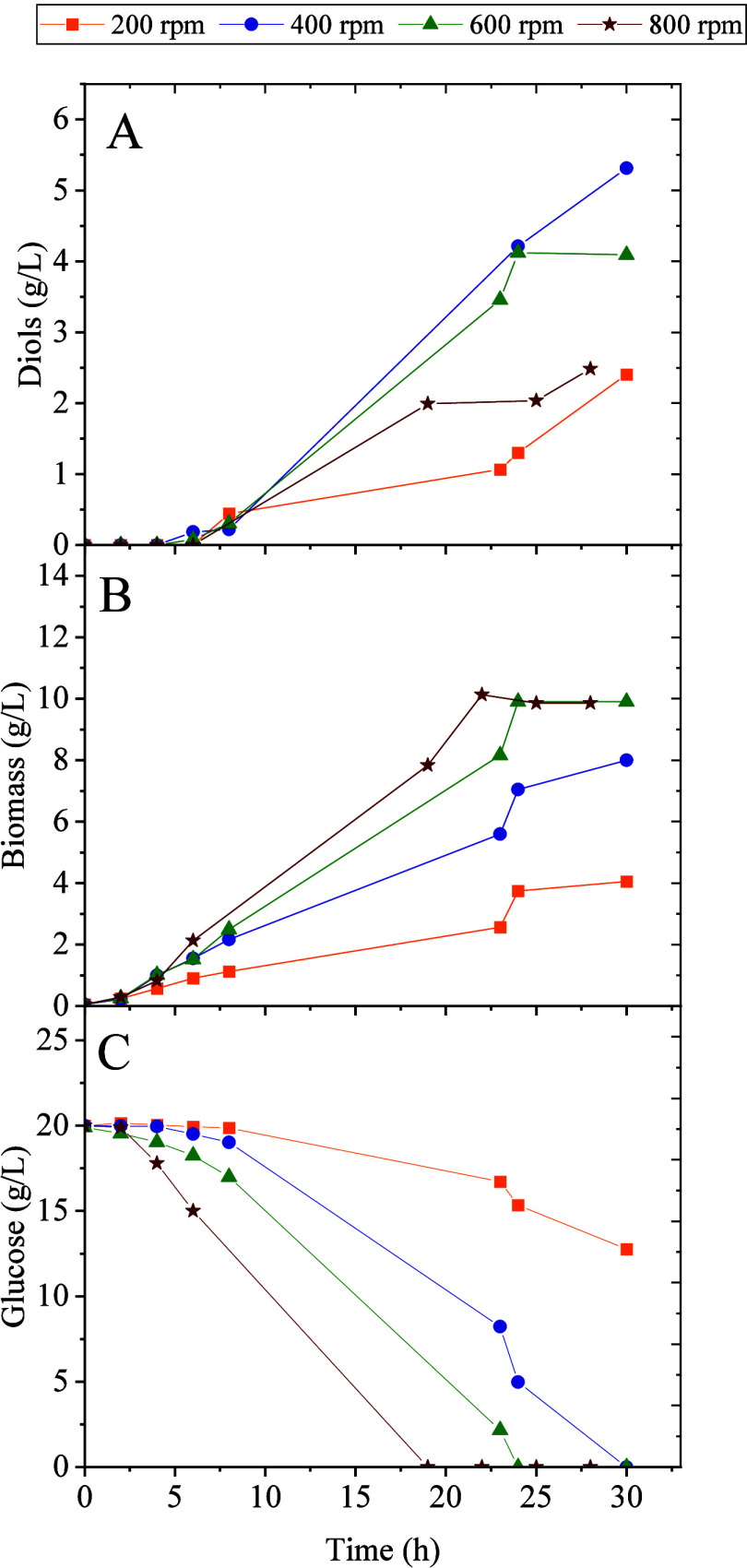
Influence of agitation in the fermentation of *P. putida* KT2440 *acoB::mini-Tn5* (pIZ2Bud) in a bioreactor with 20 g/L of glucose at different agitations. Time course of: (A) diol production; (B) biomass; and (C) glucose consumption.

The effect of the initial glucose concentration was investigated in the range of 20–100 g/L under constant agitation (400 rpm). As shown in Figure 4, *P. putida* consumed approximately 40 g/L glucose within 80 h, while diol production peaked at an initial glucose concentration of 60 g/L. However, a decline in diol production was observed when the initial glucose concentration exceeded 80 g/L. Finally, biomass evolution is affected by the initial glucose concentration, showing that the maximum biomass growth is obtained at 20 g/L of glucose. When the initial glucose concentration increases (between 40–80 g/L), the growth rate remains the same. However, at an initial glucose concentration of 100 g/L, a decrease in the growth rate is observed.

**Figure 4:**
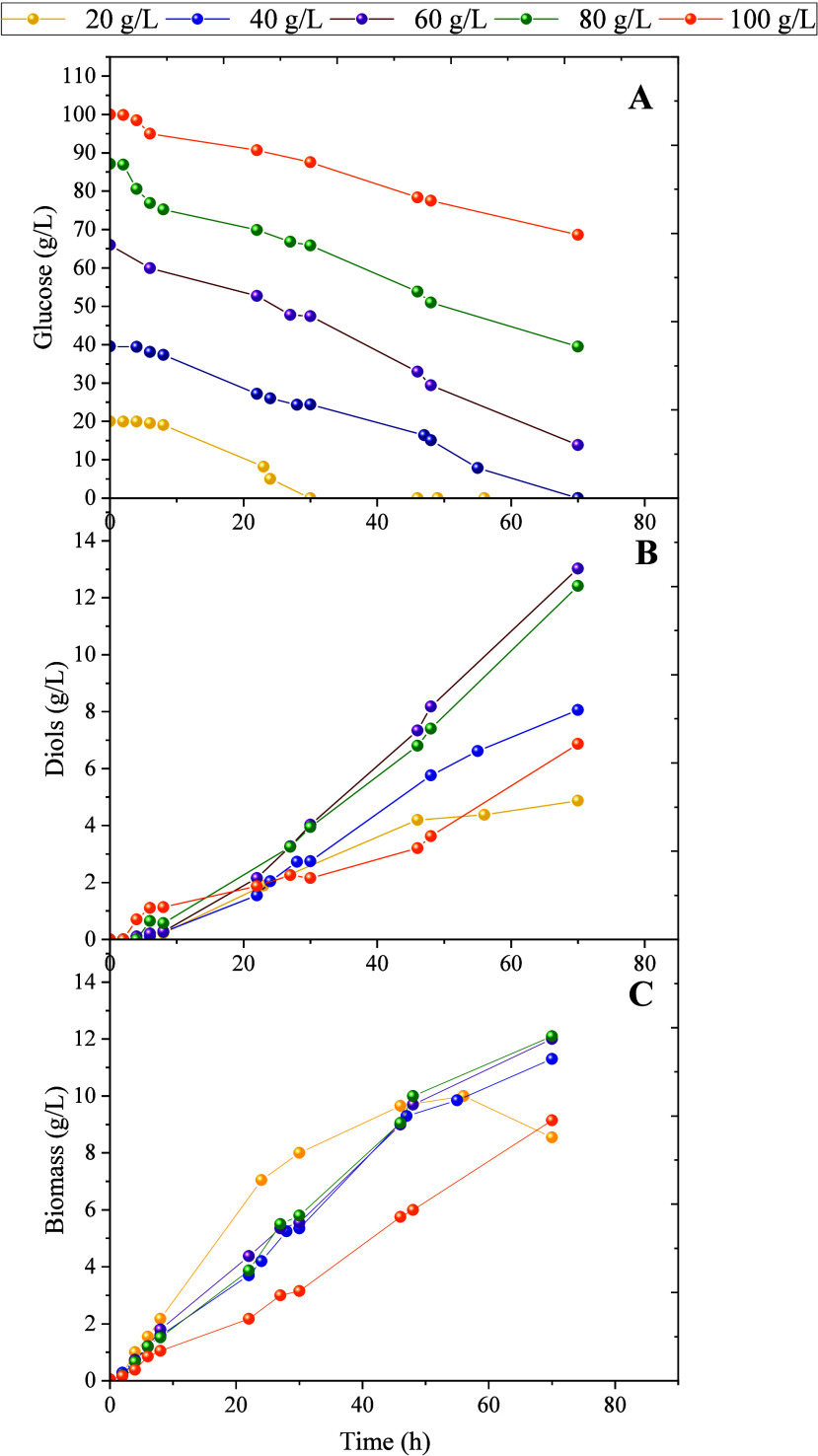
Influence of initial glucose concentration in the fermentation of *P. putida* KT2440 *acoB::mini-Tn5* (pIZ2Bud) at 400 rpm. Time course of: (A) glucose consumption; (B) diols production; and (C) biomass.

Figure 5 shows that the best yield and productivity of diols are obtained at 400 rpm; however, the selectivity decreases when the agitation increases. The same is true for the initial glucose concentration, where all parameters improve up to a glucose concentration of 40 g/L, and from this point on, the calculated parameters decrease. In the case of selectivity, we observed that this value is not influenced by the initial glucose concentration.

**Figure 5:**
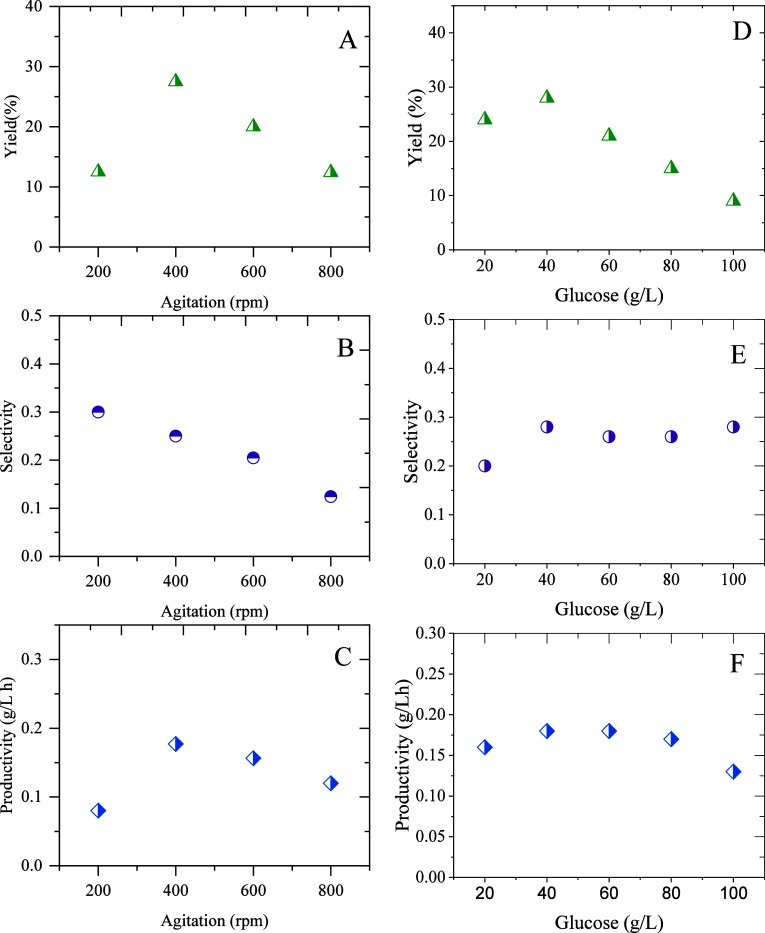
Yield (A, D), selectivity (B, E), and productivity (C, F) for the experiments performed at different agitation rates and initial glucose concentrations.

We also tested the production of 2,3-BDO under fed-batch mode, adding glucose at 40 g/L and maintaining the agitation at 400 rpm. Figure 6 shows that under these conditions, *P. putida* KT2440-*acoB::mini-Tn5* (pIZ2Bud) is able to accumulate 15 g/L of diols.

**Figure 6:**
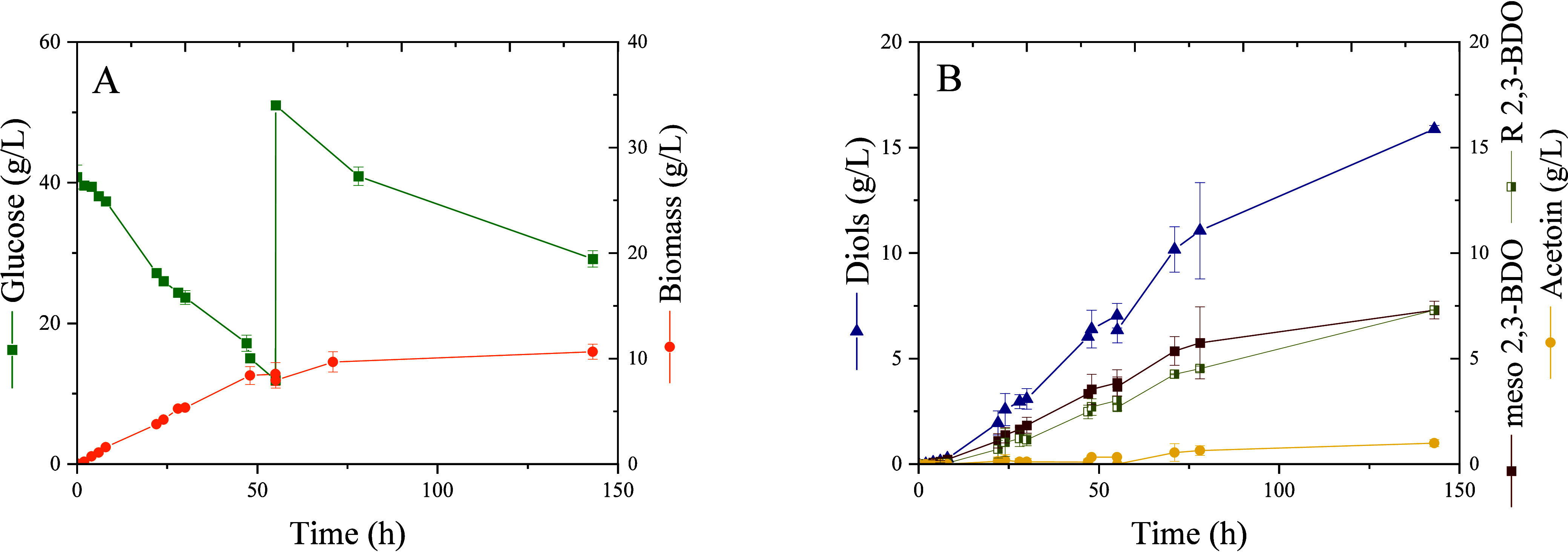
*P. putida* KT2440 *acoB::mini-Tn5* (pIZ2Bud) fed-batch fermentations in a bioreactor. (A) Evolution of glucose consumption and biomass production. (B) Production of diols (meso-2,3-BDO, R 2,3-BDO isomers, and acetoin) during the process.

Finally, we tested the production of 2,3-BDO using acetate as the carbon source. To this aim, we cultured *P. putida* KT2440*-acoB::mini-Tn5* (pIZ2Bud) in 5 g/L of acetate with different agitation speeds using a fed-batch approach (Figures 7 and 8). Although the biomass achieved at the different agitation speeds tested is very similar, the production of diols is highly dependent on the agitation speed. The recombinant strain was able to produce up to 1 g/L diols at 400 rpm, but the production decreases as the stirring speed increases.

**Figure 7:**
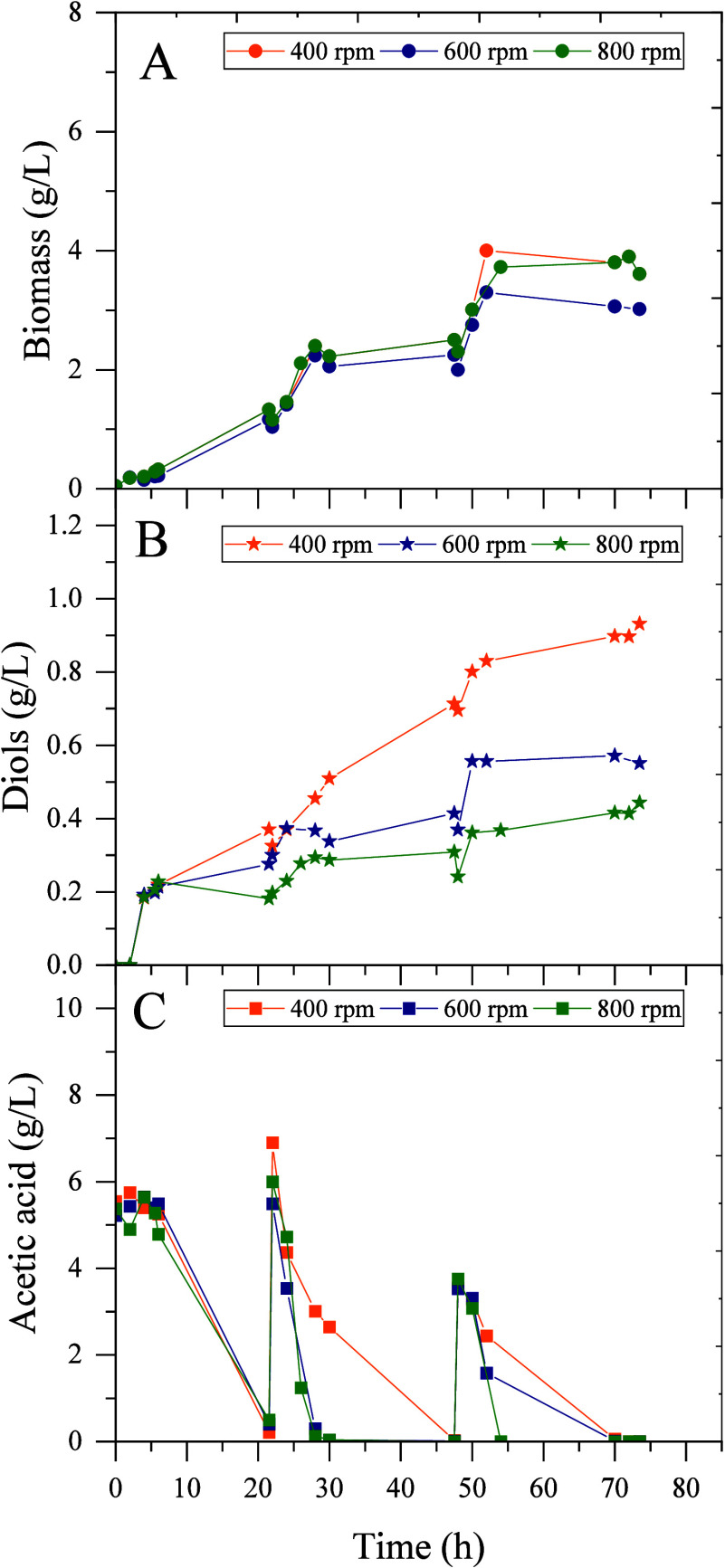
Influence of agitation in the fermentation of *P. putida* KT2440 *acoB::mini-Tn5* (pIZ2Bud) in a bioreactor with 5 g/L of acetate added in each feed at different agitation rates (400, 600, and 800 rpm). Time course of: (A) biomass; (B) diol production; and (C) acetate consumption.

**Figure 8:**
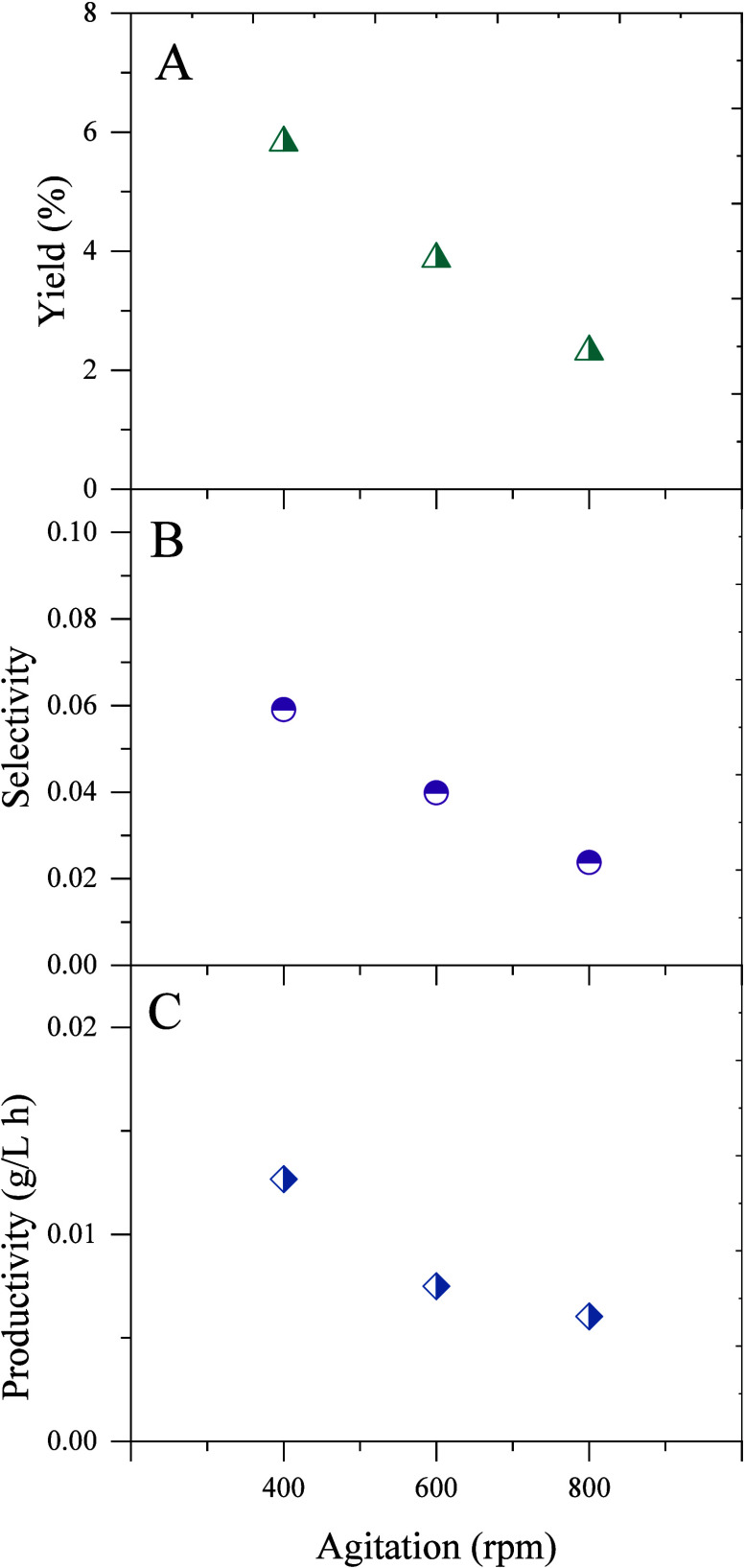
Yield (A), selectivity (B), and productivity (C) were calculated for the experiments performed in a bioreactor using different agitation rates with acetate as a substrate.

## 4 Discussion

*P. putida* KT2440 received U.S. FDA approval in 1982 as a biosafety level-1 host for recombinant DNA host–vector systems.^34^ However, this capacity to metabolize a broad range of substrates can be a drawback for producing certain compounds that can be metabolized and used as carbon sources. This is the case for some alcohols, such as n-butanol^35^ or 2,3-BDO,^19,21^ since *P. putida* KT2440 uses these alcohols and their precursors as substrates to grow. For this reason, only a few biotechnological processes have been developed in *P. putida* to produce small molecules.^36^ The only alcohol produced so far in *P. putida* was isobutanol,^37,38^ but the transformation of acetolactate into isobutanol was very low (60 mg isobutanol/g glucose) because of the low production of NADPH. In this work, we have investigated the production of 2,3-BDO because although this process also uses acetolactate as an intermediate to produce 2,3-BDO, it requires NADH instead of NADPH.

To avoid the consumption of acetoin or 2,3-BDO in *P. putida* KT2440, we tested as a putative host an *aco* mutant from a collection obtained by mini-Tn5 mutagenesis. The *P. putida* KT2440 *acoB*::mini-Tn5 mutant is defective in the E1β subunit of the acetoin dehydrogenase complex, which transforms acetoin into acetyl-CoA, and this mutant was not able to grow using acetoin or 2,3-BDO as substrates.

We have achieved the production of acetoin and 2,3-BDO in this mutant by cloning the *budABC* operon from *K. oxytoca* under the control of the IPTG-inducible *P*_*trc*_ promoter in the pIZ2 wide host range vector, which can replicate both in *P. putida* and *E. coli*. Although we cloned the BudC BDH from *K. oxytoca* that is specific for the *meso* form of 2,3-BDO, *P. putida* KT2440 *acoB*::mini-Tn5 (pIZ2Bud) produces both *meso-* and *R*-2,3-BDO (Figure S4). This finding can be explained because apart from the *budC* gene encoded in the pIZ2Bud plasmid, *P. putida* KT2440 has at least three BDHs that can use all isomers of 2,3-BDO as substrates.^39^ Therefore, in order to control the production of a specific 2,3-BDO isomer, it would be necessary to knock out the genes encoding all possible BDHs in *P. putida*.

When we tested the 2,3-BDO production in bioreactors using glucose as a substrate, we observed that the production was highly dependent on the agitation speed. The optimum agitation speed was 400 rpm because at lower speeds, cells grow poorly and produce small amounts of biomass and 2,3-BDO. However, at 200 rpm, we achieved the highest selectivity. That is, when the agitation increases, the selectivity decreases, but cell growth improves. This result can be explained by the fact that, as the transference of oxygen and carbon increases with the agitation, the substrate is funneled toward the production of biomass and CO_2_ rather than to the production of 2,3-BDO. It is known that in the presence of a high oxygen concentration, the initial precursor of 2,3-BDO, pyruvate, is mainly transformed into acetyl-CoA instead of acetolactate.^1^ Thus, the oxygen supply must be strictly regulated to balance the bacterial growth and the production of 2,3-BDO. Moreover, at the end of the fermentation, the oxygen should be reduced to avoid the reversible transformation of 2,3-BDO into acetoin when glucose is depleted from the culture medium.^40–43^

Interestingly, we determined that the initial glucose concentration plays a fundamental role in 2,3-BDO production. When the concentration of glucose is lower than 40 g/L, *P. putida* is able to consume all the substrate in less than 70 h. However, with higher glucose concentrations, this strain is unable to achieve complete substrate consumption due to an inhibitory phenomenon, which is reflected in a decrease in biomass evolution. The maximum growth rate is observed at an initial glucose concentration of 20 g/L, whereas at 100 g/L, the growth is significantly affected. However, regarding diol production, the highest values are obtained at initial glucose concentrations around 60–80 g/L. Nevertheless, at these high concentrations, the microorganism is unable to consume all the substrate, reducing the yield of the process (Figure 5D). For this reason, the highest yield is achieved at an initial glucose concentration of 40 g/L, which also coincides with the highest productivity value. Finally, carbon distribution is not influenced by the initial glucose concentration, which is reflected in the selectivity parameter, which remains unchanged.

The strategy of operating in fed-batch mode with *P. putida* KT2440 *acoB*::mini-Tn5 (pIZ2Bud) reduces substrate inhibition, improves glucose utilization, and enhances 2,3-BDO production. Under these conditions, the strain was able to produce 16 g/L of diols (0.32 g/g glucose), i.e., more than half of the theoretical maximum (0.5 g/g glucose). Interestingly, this amount of alcohols is much higher than the 3.3 g/L (0.06 g/g glucose) of isobutanol produced by the Iso2 recombinant strain of *P. putida* KT2440 ^37^ or 0.05 g/L (0.01 g/g glucose) of butanol produced by a recombinant of *P. putida* S12.^35^ The carbon contribution of the yeast extract was not considered in the yield calculations, as it contributes only to biomass formation. Including the yeast extract would result in a slightly lower yield based on total carbon.

Although higher titers and productivities have been achieved in *Klebsiella*, *Bacillus*, and *Corynebacterium* species compared to those obtained with *P. putida* in this study, the nonpathogenic nature of *Pseudomonas*, along with its ability to utilize a wide range of carbon sources, the resistance to toxic compounds, and the demonstrated potential for yield improvement through process optimization, suggest that it can be a promising host for industrial-scale 2,3-BDO production. In this sense, one of the possible substrates that could be used to produce 2,3-BDO is the hydrolysate of lignocellulosic waste. However, these hydrolysates contain certain quantities of toxic compounds, such as furfural and acetate. In this sense, *P. putida* can use acetate as a substrate and is considered a robust chassis to tolerate the presence of furfural.^44^ Here, we have compared the furfural tolerance of our two recombinant strains, demonstrating that *P. putida* is a more appropriate chassis than *E. coli* to work with hydrolysates containing high concentrations of furfural. The analyses showed that our recombinant *P. putida* strain tolerates concentrations up to 2.5 g/L, which are higher than those that would be expected in the hydrolysates, even if they are used in fed-batch processes that can concentrate the toxic compounds.

It is very interesting the behavior of *P. putida* KT2440-*acoB*::mini-Tn5 (pIZ2Bud) in acetate, since although acetate is somewhat toxic at concentrations above 5 g/L, the strain is able to use acetate to produce 2,3-BDO with a yield similar to that described for a recombinant *E. coli* 2,3-BDO producer.^32,33^ Several factors support *P. putida* as a superior microbial chassis compared to *E. coli* for acetate-based bioproduction. Unlike *E. coli*, which requires extensive metabolic reprogramming to bypass its preference for sugars, *P. putida* is evolutionarily adapted to metabolize organic acids and poor carbon sources found in diverse environments. Its robust regulatory framework—specifically the CbrA/B system and Crc protein—ensures balanced carbon flux into the TCA cycle, preventing the accumulation of inhibitory byproducts. Furthermore, as a strict aerobe, *P. putida* optimizes the ATP yield through complete acetate oxidation, enhancing biomass and product titers. Most notably, its high intrinsic tolerance to acetate-induced toxicity allows it to maintain metabolic activity under conditions that typically arrest *E. coli* growth.

The production of 2,3-BDO from acetate is limited by the metabolic and energetic constraints associated with acetate assimilation. In contrast to glucose, acetate enters the central metabolism as acetyl-CoA and must be assimilated through the glyoxylate shunt to generate C4 intermediates and pyruvate, increasing ATP demand and diverting carbon toward biomass and maintenance. Consequently, experimentally observed yields are typically well below the theoretical maximum (0.5 g g^–1^ acetate). In batch cultures, high acetate concentrations can inhibit cell growth and reduce 2,3-BDO production, whereas fed-batch strategies can mitigate this effect by maintaining noninhibitory substrate levels and improving overall carbon conversion. These aspects are particularly relevant when considering lignocellulosic hydrolysates, which commonly contain mixed substrates, such as glucose and acetate, together with inhibitors like furfural. While glucose is generally preferentially utilized, partial coassimilation of acetate may enhance carbon recovery; however, furfural detoxification imposes additional redox burdens that can further limit 2,3-BDO productivity, but our results show that *P. putida* tolerates high furfural concentrations well.

Considering the properties and robustness of *P. putida* KT2440, our results suggest that this strain may be a very useful chassis for testing 2,3-BDO production with other waste substrates that may contain some toxic substances.

## 5 Conclusions

*P. putida* KT2440 is able to produce 2,3-BDO through strain engineering of the *acoB* mutant, which prevents the consumption of acetoin and 2,3-BDO. The production is enabled by the inducible expression of the *budABC* operon from *K. oxytoca*, carried out on a plasmid. It is demonstrated that oxygen supply and initial glucose concentration must be carefully controlled to balance cell growth and 2,3-BDO synthesis. In addition, fed-batch fermentations under optimal agitation and glucose concentration enhances substrate utilization and product formation, achieving 16 g/L of diols (0.32 g/g glucose). The ability of *P. putida* to produce 2,3-BDO from acetate and to tolerate up to 2.5 g/L of furfural shows its suitability for fermentation processes using lignocellulosic hydrolysates.

## Supplementary Material



## Notes

*P. putida* KT2440 harboring the production plasmid pIZ2Bud; growth assays on 2,3-BDO and acetoin in minimal media in *P. putida* KT2440 and mutant strain *P. putida* KT2440 *aco*B::mini-Tn5; validation of 2,3-BDO production in *E. coli* DH10B (pIZ2Bud); and time-course HPLC chromatograms of 2,3-BDO and acetoin production in *P. putida* KT2440 *aco*B::mini-Tn5 with the correct IPTG concentration (PDF)
