# Supplementary material for: Engineering Pseudomonas putida for the Production of 2,3-Butanediol Isomers and Acetoin
Source: ACS Synth Biol. 2026 Apr 28;15(5):2173–83. doi: 10.1021/acssynbio.6c00243 (PMC13185156; doi:10.1021/acssynbio.6c00243)
Supplement: Supplementary file 1 [file sb6c00243_si_001.pdf]

Engineering *Pseudomonas putida* for the Production of 2,3-Butanediol Isomers and Acetoin

Irene Cano[1], Isabel de la Torre[1], Miguel G. Acedos[1,2], Jorge Barriuso[1*] and José L. Garcia[1*]

[1]Department of Biotechnology. Centro de Investigaciones Biológicas Margarita Salas. Consejo Superior de Investigaciones Científicas (CIB-CSIC). 28040 Madrid. Spain

[2]Advanced Biofuels and Bioproducts Unit, Department of Energy. Centro de Investigaciones Energéticas, Medioambientales y Tecnológicas (CIEMAT). 28040 Madrid. Spain

*Corresponding authors: Jorge Barriuso (jbarriuso@cib.csic.es) and José L. Garcia (jlgarcia@cib.csic.es)

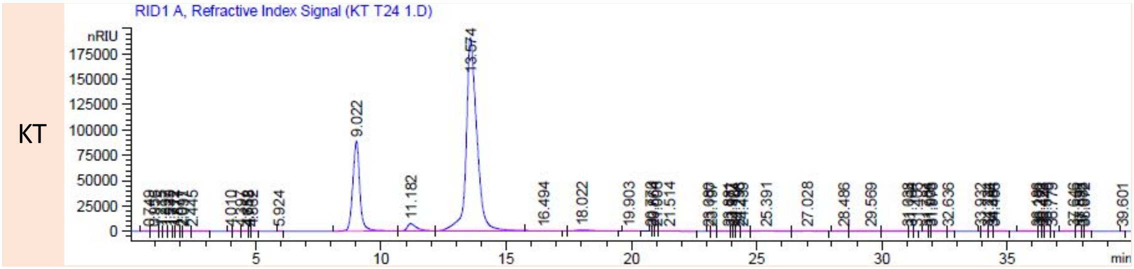

Figure S1. Example chromatogram of the *P. putida* KT2440 (pIZ2Bud) strain after 24 h of fermentation in 2,3-BDO production medium with 20 g/L of glucose showing no 2,3-BDO accumulation. The *aco* cluster is active and the 2,3-BDO produced is used as a carbon and energy source, being converted into acetyl-CoA and entering the central metabolism of the cell.

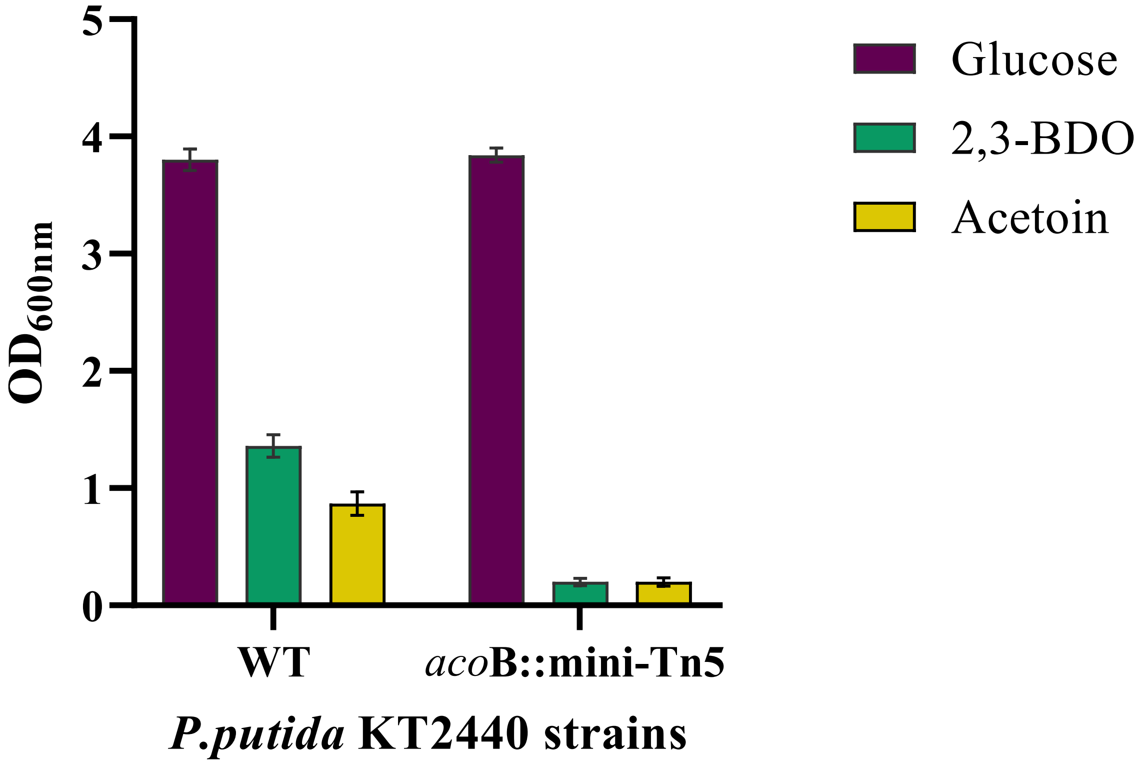

Figure S2. Growth of *P. putida* KT2440 using glucose, 2,3-BDO and acetoin. Cells of the mutant *P. putida* KT2440 (*aco*B::mini-Tn5) and *P. putida* KT2440 (wt) were cultured in M9 minimal medium supplemented with 0.2% (w/v) glucose (purple), 0.2% (w/v) 2,3-BDO, or 0.2% (w/v) acetoin (green) as the sole carbon source. Pre-cultures were grown in LB medium. Cells were harvested, washed three times with sterile saline solution (0.85% NaCl), and inoculated into minimal medium at an initial OD_{600} of 0.1 in 50 mL Erlenmeyer flasks containing 10 mL of medium. Cultures were incubated at 30 °C with orbital shaking at 200 rpm for 24 h. Growth was assessed by measuring the optical density at 600 nm.

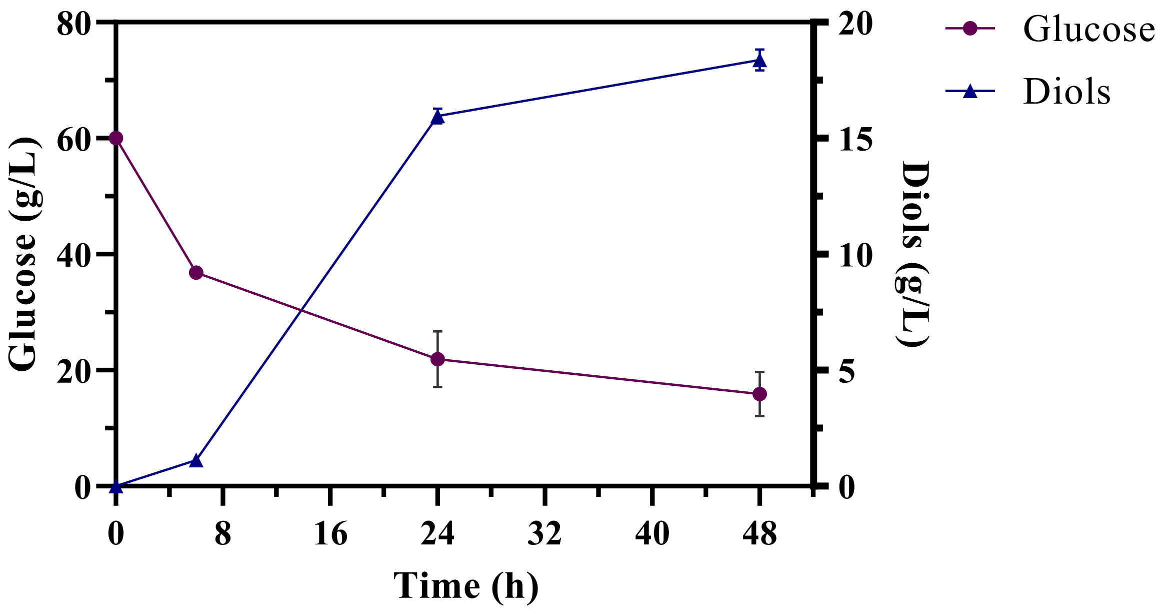

Figure S3. Production of 2,3-BDO in *E. coli*. *E. coli* DH10B (pIZ2Bud) strain was cultured in 100 mL serum bottles containing 10 mL of medium. Cultures were grown in M9YE 2× medium supplemented with 60 g/L glucose and 3 g/L yeast extract. Expression was induced with 0.5 mM IPTG after 2 h of incubation at 37 °C with orbital shaking at 200 rpm, using cotton plugs to seal the bottles. After induction, the bottles were sealed with caps and transferred to an orbital shaker at 30 °C and 200 rpm for continued incubation. Samples were taken at different times and analysed to determine the carbon source consumed and the diols produced by HPLC analysis.

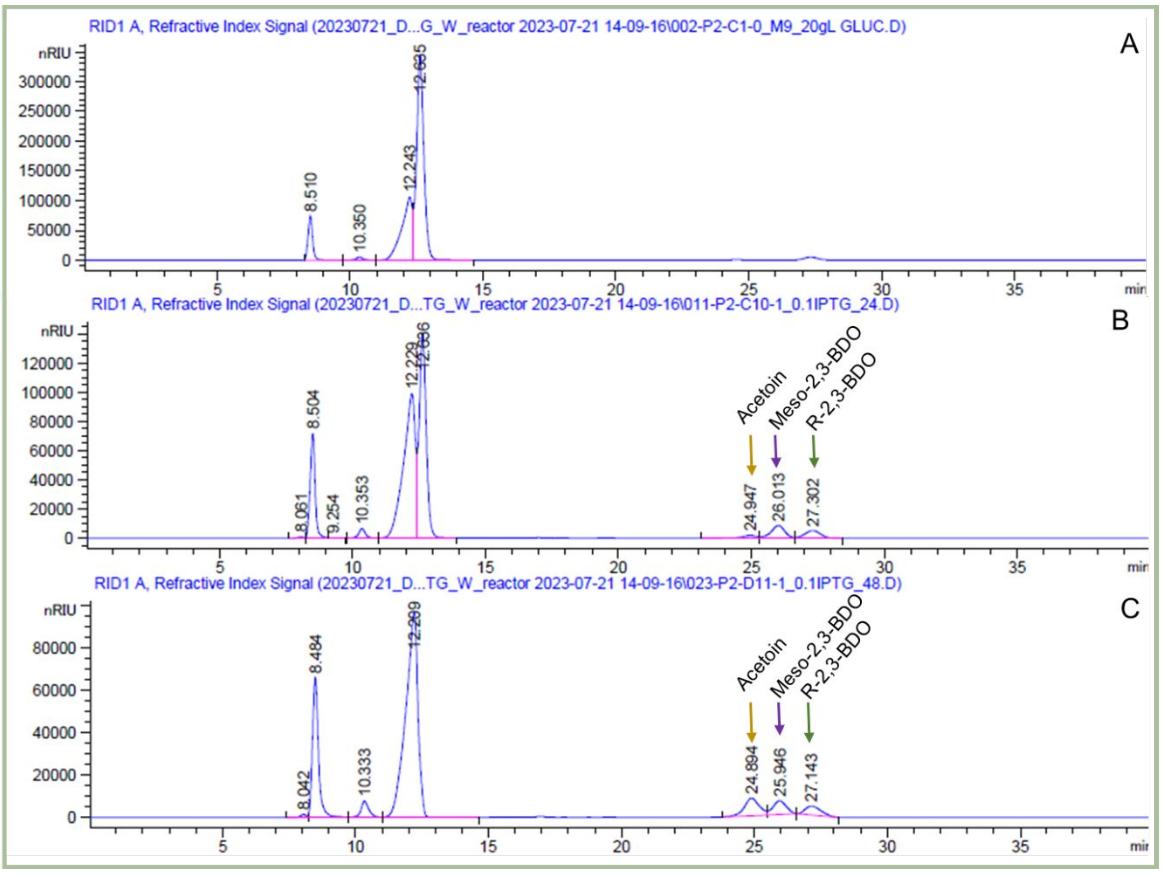

Figure S4. HPLC chromatograms of *P. putida* KT2440 *acoB::mini-Tn5* (pIZ2Bud) producing diols at initial time (A), 24 h (B) and 48 h (C). The retention time in the RID signal of each compound is indicated. The culture conditions were the ones to produce diols, using M9YE 2x supplemented with yeast extract with 20 g/L of glucose and 0.1 mM IPTG. Production and accumulation of diols are observed through the time, being accumulated both 2,3-BDO isomers and acetoin.
